# Proteins in Wonderland: The Magical World of Pressure

**DOI:** 10.3390/biology11010006

**Published:** 2021-12-21

**Authors:** Kazuyuki Akasaka, Akihiro Maeno

**Affiliations:** 1 Keihanna Academy of Science & Culture, Kansai Science City, Keihanna Interaction Plaza, Lab. Wing, Kyoto 619-0237, Japan; 2 Lab of Medical Chemistry, Kansai Medical University, 2-5-1 Shin-machi, Osaka 573-1010, Japan; maenoak@hirakata.kmu.ac.jp

**Keywords:** Anfinsen’s dogma, native state N, unfolded state U, fibril state F, protofibrils, hydrostatic pressure, hen lysozyme, circular dichroism, ^1^H NMR spectroscopy, atomic force microscopy

## Abstract

**Simple Summary:**

Unlike temperature, pH or chemical reagent, the effect of hydrostatic pressure on proteins is unique in that it controls selectively the equilibrium populations among different conformational states, without practically affecting each of their structures. Because of this unique effect of hydrostatic pressure on proteins, the application of pressure can easily manipulate the population distribution among different conformational states of a protein to a considerable extent at our will, creating what may be called “the magical world of pressure” or “the wonderland for proteins”. In this paper, we have succssfully utilized the magical world of pressure in bringing the amyloid fibrils F of wild-type hen lysozyme into the unfolded monomers U and then, upon returning to the Anfinsen regime, back into its original, natively folded state N.

**Abstract:**

Admitting the “Native”, “Unfolded” and “Fibril” states as the three basic generic states of proteins in nature, each of which is characterized with its partial molar volume, here we predict that the interconversion among these generic states N, U, F may be performed simply by making a temporal excursion into the so called “the high-pressure regime”, created artificially by putting the system under sufficiently high hydrostatic pressure, where we convert N to U and F to U, and then back to “the low-pressure regime” (the “Anfinsen regime”), where we convert U back to N (U→N). Provided that the solution conditions (temperature, pH, etc.) remain largely the same, the idea provides a general method for choosing N, U, or F of a protein, to a great extent at will, assisted by the proper use of the external perturbation pressure. A successful experiment is demonstrated for the case of hen lysozyme, for which the amyloid fibril state F prepared at 1 bar is turned almost fully back into its original native state N at 1 bar by going through the “the high-pressure regime”. The outstanding simplicity and effectiveness of pressure in controlling the conformational state of a protein are expected to have a wide variety of applications both in basic and applied bioscience in the future.

## 1. Introduction

Thermodynamics is a fundamental branch of physics that all creatures on earth must obey to live. The well-known Anfinsen’s dogma [1], governing the fate of a newborn polypeptide chain “U” to fold into a unique conformation “N” in an aqueous environment under physiological conditions, is a type of this example. Although certain proteins may need some help from other proteins called chaperones for folding, this process is not considered to alter the essence of Anfinsen’s dogma. As such, the “Anfinsen’s dogma” may possibly have prevailed on the earthy atmosphere for billions of years of evolution to bring up all (~10 million) archaea, prokaryote and eukaryote species considered to be living presently on earth [2]. On the other hand, by taking Anfinsen’s dogma more generally from the thermodynamic and statistical mechanical viewpoint, we can view the unfolding and refolding process of protein as a shift of the conformational distribution induced by thermodynamic perturbation in an aqueous environment [3]. Here, pressure is a particularly important thermodynamic variable besides temperature and solution conditions in controlling the conformational state of a protein in an aqueous environment.

Experimental studies on the conformational state of proteins under pressure perturbation started early in the 1980s when Hui Bon Hoa contributed greatly to advancing various spectroscopic techniques under pressure and promoted their applications [4,5,6]. Protein high-pressure NMR spectroscopy, re-initiated by Jonas in the 1990s [7], was expanded into a crucial methodology to probe the fluctuating nature of protein structure in later years [8,9,10,11,12]. The method disclosed the notion that, in most globular proteins, the unfolded conformer U is always present in equilibrium with the folded conformer N, even under physiological conditions at 1 bar [8,9].

In accordance with this, an additional state of a protein sometimes called the “misfolded state” [13] has come to gain attention in a wide range of protein systems. It turns out that many of them grow into misfolded oligomers [14], which then turn into a fibrillar state commonly called “protofibrils”, which consist of single or a few lines of a linear array of “unfolded” protein monomers forming “the cross β-structure”, detailed features of which may vary from protein to protein [15,16]. Importantly, they are known to be the primary cause of a variety of human diseases known as “amyloidosis disease” including Alzheimer disease [17], familial amyloidotic polyneuropathy [18], transthyretin amyloidosis [19], prion disease [20], dialysis-related amyloidosis [21], alpha-synuclein amyloidosis [22] and senile systemic amyloidosis [23] and so on. Furthermore, as it is becoming increasingly clear that the propensity to form amyloid fibrils is a generic property for all proteins including food proteins, protein fibrillization is progressively recognized in food science as a strategy to broaden and improve food protein functionality [24].

Many of the amyloid diseases above arise from single mutations in their wild-type proteins, which often destabilize the N state and increase the equilibrium population of the U state; for example, we previously found in the V30M mutant of transthyretin, that the population of the U conformer increased by as much as 1000-fold, causing fatal amyloidosis [25]. The important point here is that, despite the fact that the protein obeys Anfinsen’s dogma to fold, an equilibrium fraction of the protein may always remain unfolded in the cell environment. Then, a crucial question may remain as to how the unfolded state U may turn into the fibrillar state F when there is no substance to catalyze the reaction.

To answer this question, a model study was carried out previously [26], by using an authentic, fully (~100%) unfolded conformer of a protein, prepared from ^15^N-labeled wild-type hen lysozyme with its four disulfide bonds removed (0SS mutant), to see if they would turn into a protofibril at all and if so, how would it proceed? We found that the protofibrils are formed simply by a prolonged incubation of its aqueous solution over several days at 25 °C at 1 bar [26]. We also found that the protofibrils so formed can then be dissociated back into the monomeric state U (U→F→U) nearly completely simply by applying pressure at 2 kbar for ~50 h [27]. Detailed analysis indicates that the reaction proceeds by a simple reaction, with monomers attached and/or detached at its growing end of the fibril [28]. We believe that the basic mechanisms disclosed here for the simplest system will form the basis for understanding the reality of the protofibril formation and dissociation U→F→U in most amyloidotic proteins.

**Figure 1:**
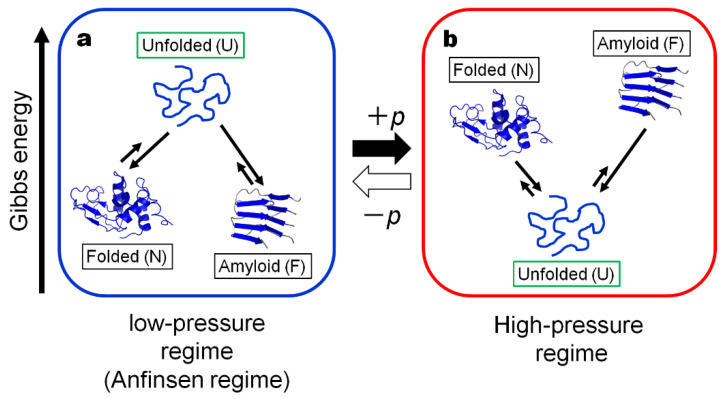
The schematic Gibbs energy diagrams for the proposed three generic forms of a protein; Folded (N), Unfolded (U) and Amyloid fibril (F): (**a**) in the “Anfinsen regime” or the low-pressure regime and (**b**) in the high-pressure regime. The “Anfinsen regime” is the regime realized in nature, while the high-pressure regime is the regime artificially realized in high-pressure vessels. Note that the thermodynamic stabilities of N, U and F are reversed in the two regimes, because of the large *p*Δ*V* contribution. Here the relative Gibbs energy levels for U, N and F are drawn arbitrarily.

Under the condition, the concept of the protein conformational regime under physiological conditions at low pressure (“Anfinsen regime”) may be extended to include the amyloid fibril state “F” as another generic state of a globular protein in the low-pressure regime (or the Anfinsen regime), basically in dynamic equilibrium with the U and N states as schematically shown in Figure 1a. Here we define state F to be represented by “protofibrils”, rather than the “matured amyloid fibrils” which may contain the insoluble entity.

An important property of F as well as of N is that they both are in a high-volume state [29], as they generally have extra space or “vacancy” (called void, cavity, or defects) within their basic architecture, while the state U exists in a low-volume state with most of the extra “vacancies” gone, leaving the polypeptide chain in direct contact with the bulk water. Because of these contrasting properties between U and N–F, their relative thermodynamic stabilities are anticipated to be reversed rather easily by applying a sufficiently high pressure (a few to several kbar).

Indeed, under a high hydrostatic pressure far exceeding 1 kbar (>1 kbar), both the folded conformation N and the fibril state F become less stable than U, fully reversing the order of thermodynamic stability predicted from Anfinsen’s dogma in the low-pressure regime, namely U is more stable than N and F at elevated pressures, thereby forming what we call here “the high-pressure regime” (See Figure 1b). Note that “the high-pressure regime” is realizable relatively easily nowadays in high-pressure vessels in scientific laboratories and possibly in food industries where pressures up to several kbar are regularly used.

## 2. Thermodynamic Consideration

### 2.1. In the “Anfinsen Regime” or the Low-Pressure Regime 

The well-known Anfinsen’s dogma [1], governing the fate of a newly born polypeptide chain U to fold into a unique conformation N, states in essence that, for most proteins in nature under physiological conditions (~at 1 bar), the Gibbs energy *G*_U_^0^ for the unfolded state U is higher than the Gibbs energy *G*_N_^0^ for the folded state N, namely, Δ*G*_1bar_ = Δ*G*^0^ = *G*_U_^0^ − *G*_N_^0^ > 0, pushing the unfolded polypeptide chain “U” toward the folded state “N”. Here for most globular proteins in nature, Δ*G*_1bar_ = Δ*G*^0^ takes a small, but the positive value at 1 bar, often within a few tens of kJ/mol, leaving the state N marginally stable against the state U. Here we postulate “amyloid fibril state F” represented by protofibrils to be another generic state of a protein stable at low pressure, giving another minimum in free energy besides the native state N in the “Anfinsen regime”.

### 2.2. In the “High-Pressure Regime”

At high pressure *p*, the thermodynamic stability of N against U, Δ*G*(*p*), is given approximately to the first order in *p* by Equation (1),

Δ*G*(*p*) = *G*_U_(*p*) − *G*_N_(*p*) = Δ*G*^0^ + Δ*V* (*p* − *p*^0^) (1)

Here the volume difference Δ*V* between U and N (Δ*V* = *V*_U_ − *V*_N_) is always negative under or below physiological temperatures [8], making the term Δ*V* (*p* − *p*^0^) take a large negative value for large *p*. The high-volume nature of N against U has been well documented in many proteins. Then at high pressure, the negative Δ*V* (*p* − *p*^0^) term (<0) exceeds in magnitude the positive Δ*G*^0^ term (>0), resulting in a negative Δ*G* = *G*_U_ − *G*_N_ (<0), meaning that N turns into U causing “pressure-denaturation” of a protein.

In the case of a typical globular protein of a few kDa in size, the Δ*V* could be ~−100 mL/mol, making *p*Δ*V* to be ~−20 kJ/mol at 2 kbar and ~−30 kJ/mol at 3 kbar. If Δ*G*^0^, the excess stability of N over U at 1 bar, is typically ~15 kJ/mol, Δ*G*(*p*) in Equation 1 would become ~−5 kJ/mol at 2 kbar and ~−15 kJ/mol at 3 kbar, replacing conformer N (~99.9%) at 1 bar by conformer U (>90%) at 2 kbar, (>99%) at 3 kbar, namely the relative stability of N and U in the Anfinsen regime is likely to be almost fully reversed in the “high-pressure regime” (Figure 1b). When pressure is removed (back to the Anfinsen regime), the state N will recover quickly and reversibly to the original population close to ~99%. Basically, such reversibility has been recognized in most globular proteins including RNase A, hen lysozyme, Staphylococcal nuclease, Apo myoglobin, β-lactoglobulin, P13MTCP1, transthyretin, prion proteins, and ubiquitin, at physiological temperatures or lower [9].

In the case of an amyloid fibril, its high-volume nature was revealed by the results of direct time-dependent densitometry measurement of protofibrils (F) upon its formation from (U) in the intrinsically denatured disulfide-deficient hen lysozyme, which showed a large volume increase by as much as Δ*V* ~ 570 mL/mol monomer U [29]. The result suggests that the formation of F from U involves a formation of large voids or cavities and/or ionic bonds, in analogy to, or even more than the case for, the formation of N from U. Granting that a high volume is a general property of any amyloid fibrils, pressure is expected to destabilize F significantly against U (ΔG = G_U_ − G_F_ < 0), because of the large negative *p*Δ*V* term in Equation (1). Now we can depict a schematic free energy diagram for F, N and U at high pressure in Figure 1b, where the relative stability of F, N and U is reversed from that in the Anfinsen regime at low pressure, the relative stability of N and F depends on the protein concentration among other factors. We may call the regime represented by the scheme in Figure 1b the “high-pressure-regime”, which is a fully artificial regime of protein thermodynamics that apparently has not been experienced by most living creatures in their history on earth.

Thus, by switching between the “high-pressure-regime” and the “Anfinsen” regime simply by turning the pressure on and off, we would get a magical power for selecting the conformational state of a protein among N, F and U to a great extent at will. A successful experiment in freely controlling the conformational state among N, F and U will be demonstrated below in the experiment with wild-type hen lysozyme as representative. Here we will demonstrate the nearly full conversion (>90%) of the fibrils (F) back into the folded state (N), simply by temporarily visiting “the magical world of pressure” (the high-pressure regime) and back to the Anfinsen regime (the low-pressure regime).

## 3. Materials and Methods

Preparation of amyloid fibrils: Lyophilized hen egg white lysozyme (crystallized six-times) was purchased from Seikagaku Kogyo (Tokyo, Japan) and used without further purification. To prepare the amyloid fibrils consisting largely of intact (i.e., non-degraded) hen lysozyme, we used the “seeding method” reported previously in the literature [30]. This seeding method is briefly mentioned as follows. As the first step, the powder of wild type hen lysozyme was dissolved to a final concentration of 8.0 mg/mL in distilled water containing 80 mM NaCl with pH adjusted to 2.2 by HCl, and the solution was incubated at 57 °C, which is close to the transition temperature of hen lysozyme. In the association process to amyloid fibrils of hen lysozyme, the exponential increase in thioflavin T (Th T) fluorescence intensity with increasing incubation time was observed (Figure S1a). After ~11 days, the resultant amyloid fibrils as the first generation was subjected to extensive sonication to produce oligomers, an aliquot of which (10 *w*/*w* %) was mixed into a fresh solution of 90 *w*/*w* % of intact hen lysozyme (8.0 mg/mL in 80 mM NaCl at pH 2.2) and incubated for 4.5 h at 57 °C. The second-generation fibrils produced in this way were subjected to extensive sonication to produce seeds. Then, an aliquot of these seeds (4 *w*/*w* %) was mixed with a fresh solution of 96 *w*/*w* % of intact hen lysozyme (8.0 mg/mL in 80 mM NaCl at pH 2.2) and incubated for 4.5 h at 57 °C to produce the third-generation fibrils consisting almost entirely of intact hen lysozyme molecules, supported by the analysis of SDS-PAGE showing the non-degradation of intact hen lysozyme in the period of fibrillation process (Figure S1b).

Dissociation of amyloid fibrils with pressure: The fibril solution of the third-generation was diluted by 20-fold to produce a fibril solution, containing 0.40 mg/mL fibrils in 80 mM NaCl, pH adjusted to 2.2. This fibril solution was then placed in a homemade Teflon inner vessel (volume~100 μL) and was pressurized at 4 kbar in a pressuring machine Dr. CHEF (KOBELCO, Kobe, Japan) for a varied reaction time between 0.5 and 24 h at 25 °C. After each reaction time, the fibril solution was subjected to the circular dichroism (CD) and ^1^H NMR measurements without further dilution, while AFM measurements were made after dilution and drying by air on a silica surface. The ^1^H NMR spectra were measured at 25 °C at 600 MHz on an AVANCE 600 NMR spectrometer (Bruker Biospin, Switzerland) using a standard 5 mm outer diameter tube (Shigemi, Tokyo, Japan). The CD spectra in the far UV region were measured at 25 °C on a J-820 spectropolarimeter (JASCO Co., Tokyo, Japan) using a sample cell of 0.1 cm light path after 1 h of pressure-treatment. The AFM images were obtained at 1 bar at 25 °C with the cyclic contact mode at a frequency of 119 kHz on an SPI-3800 (Seiko Instruments Inc., Tokyo, Japan).

## 4. Results

### 4.1. Experimental Demonstration: Turning Amyloid Fibrils “F” Back into the Folded State “N” in Hen Lysozyme

Then, by taking a well-known globular protein hen lysozyme as an example, we will present an actual experimental demonstrating the full conversion of hen lysozyme amyloid fibrils F into the original folded native conformation N by going through the “high-pressure regime”—the magical world of pressure.

The experimental procedure:

(1) N→U→F conversion in the Anfinsen regime: First, in the Anfinsen regime (at 1 bar), we convert the folded conformer N into the unfolded conformer U by heating hen lysozyme solution (8.0 mg/mL, pH 2.2) close to its transition temperature 57 °C at pH 2.2, and then convert U into amyloid fibrils F by seeding with lysozyme oligomers (see Materials and methods). The seeding procedure is repeated to prepare the final fibrils consisting almost exclusively of intact (non-degraded) hen lysozyme molecules.

(2) F→U conversion in the “high-pressure regime”: Then, after diluting the fibril solution by 20-fold, we switch to the “high-pressure regime” by applying pressure at 4 kbar at 25 °C and turn the fibrils F into U (actually a mixture of a one-to-one ratio of N and U at 4 kbar) at 25 °C. The process of dissociation of the fibrils with a time of exposure to 4 kbar was step-wise monitored at 1 bar with AFM and ^1^H NMR.

(3) U→N conversion in the Anfinsen regime: Then by lowering pressure to 1 bar, we switch back to the Anfinsen regime and complete the folding of U back to the original native conformer N at 25 °C (U→N conversion in the Anfinsen regime). The 20-fold dilution assures the pathway U to N rather than the pathway U to F, depending on the protein concentration, in the Anfinsen regime. Thus the entire conformational cycle of hen lysozyme, N→(U)→F→(U)→N, is to be completed.

### 4.2. The Experimental Result

**Figure 2:**
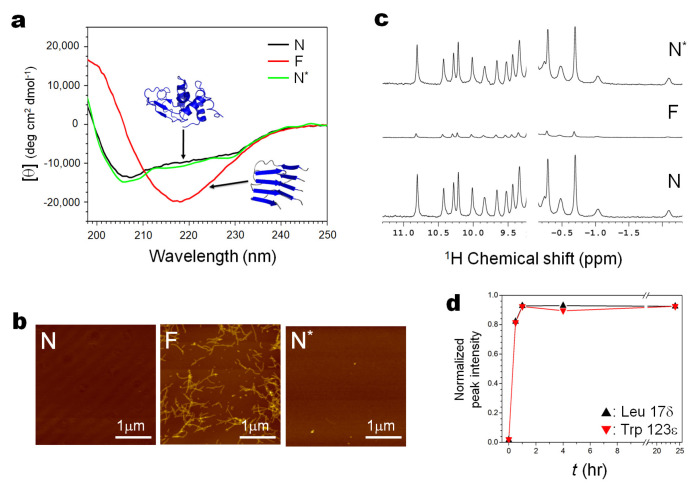
The conversion of the folded form (N) of wild-type hen lysozyme into the amyloid fibril form (F) and then refolding back to the folded form (N), by following the process N→(U)→F→(U)→N*. The experiments are monitored by CD (**a**), AFM (**b**) and ^1^H NMR (**c**,**d**), all at 1 bar at 25 °C. (The refolded N is designated as N*, showing that it is a refolded N, although their conformations are identical). See Methods for detailed experimental procedures.

Figure 2 summarizes the result of the experiment, which starts from N, goes into F, and then returns back to N, along the arrows N→(U)→F→(U)→N* (the asterisk * is to indicate a refolded N, through N and N* should be identical).

Here Figure 2a demonstrates the result with CD as a monitor for both fibrils and monomers, Figure 2b demonstrates the result with AFM as a monitor for fibrils, and Figure 2c demonstrates the result with ^1^H NMR as a monitor for dissociated monomers from fibrils, all measured at 1 bar at 25 °C.

The far-UV CD spectra (Figure 2a) show that N, showing a characteristic α-rich structure (~30% of α-helix and ~20% of β-sheet) with a minimum at ~208 nm, is converted with an iso-dichroic point at ~211 nm to F, showing a characteristic β-rich structure with a minimum at ~218 nm, and back to the original N nearly completely with an iso-dichroic point at ~211 nm. The AFM figures (only detectable of fibrils) (Figure 2b) start from N with no images, then to F showing typical amyloid fibrils, and then back to N with all fibril images gone, consistent with the observation with CD. The ^1^H NMR spectra (only detectable of monomers) start from the well-known N signals of hen lysozyme (~100%) (Figure 2c), and is converted to “F signals”, actually a trace of the N signals that remain in equilibrium with F, and back to the N* signals showing nearly a full refolding into N (>90%). The time-dependent ^1^H NMR signals (Figure 2d), monitored intermittently at 1 bar, after exposure of the fibril solution to 4 kbar for a certain period of time, show that the dissociation of the fibrils starts rapidly and is nearly complete within 1 h.

(Figure 2a) N→(U)→F→(U)→N* with CD as a monitor for both fibrils (F) and folded monomers (N). The far-UV CD spectra show that N, showing a characteristic β-rich structure with a minimum at ~208 nm (black), is converted with an iso-dichroic point at ~211 nm to F by seeding at 57 °C, showing a characteristic β-rich structure with a minimum at ~218 nm (red), and back to the original N nearly completely with an iso-dichroic point at ~211 nm (green).

(Figure 2b) N→(U)→F→(U)→N* with AFM as a monitor for fibrils (F). The AFM figures (only detectable of fibrils) start from N with no images, then to F showing typical amyloid fibrils, and then back to N with all fibril images gone, consistent with the observation with CD. 

(Figure 2c) N→(U)→F→(U)→N* with ^1^H NMR as a monitor for dissociated monomers. The ^1^H NMR spectrum (only detectable of monomers) with all NMR peaks characteristic for N of hen lysozyme (~ 100% of an intact protein), is converted to “F” showing only a trace of signals of N (in equilibrium with N) and back to N* (after treatment for 24 h at 4 kbar at 25 °C) showing nearly full refolding into N (>90%).

(Figure 2d) F→(U)→N*, monitoring the dissociation of fibrils with the time-dependent ^1^H NMR signals of the dissociated monomers, as the fibril solution is treated at 4 kbar at 25 °C for respective time intervals. The result shows that the dissociation is completed within 2 h.

## 5. Discussion

The example above demonstrates that we can, in fact, choose the conformational state of a protein among N, U and F in a simple and logical way, by temporarily visiting the “high-pressure regime”, or “the magical world of pressure”, which is surely realizable in high-pressure vessels, and by coming back to the Anfinsen regime to finish the dream. The high-pressure regime is an artificially created “WONDERLAND for protein” in which the relative conformational stability among N, F and U is largely reversed from that in the Anfinsen regime. Choice of the regime between the so-called “Anfinsen” and the “high-pressure” regime can be made simply by “switching pressure off or on” at an appropriate level of pressure.

An increasing number of proteins of physiological relevance have now been recognized as causing amyloidosis, a major hazard to human health in recent years. The formation of amyloidosis fibrils in vivo occurs as an irreversible process in a long time-range and is thought to be the cause of many hitherto incurable human diseases. However, as we have seen here, in a shorter time range, the amyloid fibril formation starts with the formation of protofibrils, which are in equilibrium with their monomeric counterpart and are dissociable under pressure. Thus, the combined use of the low pressure “Anfinsen” and the “high-pressure” regime as shown here, in the early phase of the disease, should have a potential utility in preventing and/or curing amyloid diseases. For the case of food-based amyloidosis, one promising example has been carried out for the fibrils of a prion disease, which are not only dissociated but efficiently degraded by proteinase K under pressure [31]. The result may promise some industrial and biomedical applications in the future. For the case of amyloidosis in human tissues, we might need to invent some new technologies for pressurizing a selective portion of the tissue or the fluid directly. Otherwise, the target tissue or fluid could be temporarily removed from the patient’s body and returned after treatment with pressure, as partly realized for the eradication of malignant melanoma [32].

## 6. Conclusions

The “Anfinsen regime” where the “Anfinsen’s dogma” operates has been a reality dominating the current biological world, assuring the prosperity and versatility for all lives on earth including humans, though with a constant danger of the proteins going into stable aggregates with no function. In contrast, the “high-pressure regime” proposed here as a counter regime is also a reality only realized at present in high-pressure vessels in scientific laboratories, in which proteins could temporarily escape from the burden of the Anfinsen’s dogma. Through this regime, however, one can have a magical power of freeing the protein from forming amyloid fibrils and then back to the “Anfinsen regime” to promote folding back into the N state. The entire process from N to U to F and back from F to U to N has been successfully demonstrated here in the experiment with wild-type hen lysozyme as a model protein. To realize our dreams further into bio-scientific reality, our traveling into the wonderland of pressure will continue.

## Data Availability

Not applicable.

## Supplementary Materials

The following is available online at: https://www.mdpi.com/article/10.3390/biology11010006/s1. Figure S1: (a) The NaCl concentration-dependent fibrillation process of 4.0 mg/mL hen lysozyme with 4%(*w*/*w*) seeds at pH 2.2 at 57 °C, monitored by thioflavin T fluorescence at 482 nm for the varied NaCl concentration of 4, 10, 20, 40 and 80 mM. (b) The SDS-PAGE analysis of the products produced in the fibrillation reaction of wild-type hen lysozyme (4.0 mg/mL) in the presence of 4% (*w*/*w*) seeds in 80 mM NaCl, at pH 2.2 at 57 °C (cf. (a)) for the incubation times 0–10 h. Lane M is for molecular weight markers and Lane C is for authentic hen lysozyme (14.4 kDa).
