# Supplementary material for: Proteins in Wonderland: The Magical World of Pressure
Source: Biology (Basel). 2021 Dec 21;11(1):6. doi: 10.3390/biology11010006 (PMC8772990; doi:10.3390/biology11010006)
Supplement: Supplementary file 1 [file biology-11-00006-s001.zip › biology-1446395-supplementary.pdf]

biology

Supplementary Materials

Proteins in Wonderland: The Magical World of Pressure

Kazuyuki Akasaka and Akihiro Maeno

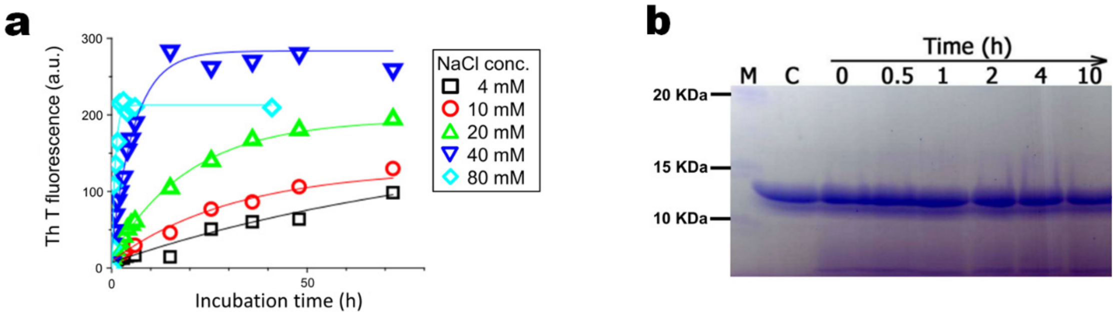

Figure S1. (**a**) The NaCl concentration-dependent fibrillation process of 4.0 mg/ml hen lysozyme with 4 %(*w*/*w*) seeds at pH 2.2 at 57 °C, monitored by thioflavin T fluorescence at 482 nm. for the varied NaCl concentration of 4, 10, 20, 40 and 80 mM. (**b**) The SDS-PAGE analysis of the products produced in the fibrillation reaction of wild-type hen lysozyme (4.0 mg/ml) in the presence of 4 %(*w*/*w*) seeds in 80 mM NaCl, at pH 2.2 at 57 °C (cf. (a)) for the incubation times 0-10 h. Lane M is for molecular weight markers and Lane C is for authentic hen lysozyme (14.4 kDa).
